# Association of Disparities in Family History and Family Cancer History in the Electronic Health Record With Sex, Race, Hispanic or Latino Ethnicity, and Language Preference in 2 Large US Health Care Systems

**DOI:** 10.1001/jamanetworkopen.2022.34574

**Published:** 2022-10-04

**Authors:** Daniel Chavez-Yenter, Melody S. Goodman, Yuyu Chen, Xiangying Chu, Richard L. Bradshaw, Rachelle Lorenz Chambers, Priscilla A. Chan, Brianne M. Daly, Michael Flynn, Amanda Gammon, Rachel Hess, Cecelia Kessler, Wendy K. Kohlmann, Devin M. Mann, Rachel Monahan, Sara Peel, Kensaku Kawamoto, Guilherme Del Fiol, Meenakshi Sigireddi, Saundra S. Buys, Ophira Ginsburg, Kimberly A. Kaphingst

**Affiliations:** 1 Huntsman Cancer Institute, University of Utah, Salt Lake City; 2 Department of Communication, University of Utah, Salt Lake City; 3 School of Global Public Health, New York University, New York, New York; 4 Department of Biomedical Informatics, University of Utah, Salt Lake City; 5 School of Medicine, University of Utah Health, Salt Lake City, Utah; 6 Perlmutter Cancer Center, NYU Langone Health, New York, New York; 7 Department of Population Health Sciences, University of Utah, Salt Lake City; 8 Department of Internal Medicine, University of Utah, Salt Lake City; 9 Department of Population Health, New York University Grossman School of Medicine, New York University, New York, New York; 10 Center for Global Health, National Cancer Institute, Rockville, Maryland

## Abstract

**Importance:**

Clinical decision support (CDS) algorithms are increasingly being implemented in health care systems to identify patients for specialty care. However, systematic differences in missingness of electronic health record (EHR) data may lead to disparities in identification by CDS algorithms.

**Objective:**

To examine the availability and comprehensiveness of cancer family history information (FHI) in patients’ EHRs by sex, race, Hispanic or Latino ethnicity, and language preference in 2 large health care systems in 2021.

**Design, Setting, and Participants:**

This retrospective EHR quality improvement study used EHR data from 2 health care systems: University of Utah Health (UHealth) and NYU Langone Health (NYULH). Participants included patients aged 25 to 60 years who had a primary care appointment in the previous 3 years. Data were collected or abstracted from the EHR from December 10, 2020, to October 31, 2021, and analyzed from June 15 to October 31, 2021.

**Exposures:**

Prior collection of cancer FHI in primary care settings.

**Main Outcomes and Measures:**

Availability was defined as having any FHI and any cancer FHI in the EHR and was examined at the patient level. Comprehensiveness was defined as whether a cancer family history observation in the EHR specified the type of cancer diagnosed in a family member, the relationship of the family member to the patient, and the age at onset for the family member and was examined at the observation level.

**Results:**

Among 144 484 patients in the UHealth system, 53.6% were women; 74.4% were non-Hispanic or non-Latino and 67.6% were White; and 83.0% had an English language preference. Among 377 621 patients in the NYULH system, 55.3% were women; 63.2% were non-Hispanic or non-Latino, and 55.3% were White; and 89.9% had an English language preference. Patients from historically medically undeserved groups—specifically, Black vs White patients (UHealth: 17.3% [95% CI, 16.1%-18.6%] vs 42.8% [95% CI, 42.5%-43.1%]; NYULH: 24.4% [95% CI, 24.0%-24.8%] vs 33.8% [95% CI, 33.6%-34.0%]), Hispanic or Latino vs non-Hispanic or non-Latino patients (UHealth: 27.2% [95% CI, 26.5%-27.8%] vs 40.2% [95% CI, 39.9%-40.5%]; NYULH: 24.4% [95% CI, 24.1%-24.7%] vs 31.6% [95% CI, 31.4%-31.8%]), Spanish-speaking vs English-speaking patients (UHealth: 18.4% [95% CI, 17.2%-19.1%] vs 40.0% [95% CI, 39.7%-40.3%]; NYULH: 15.1% [95% CI, 14.6%-15.6%] vs 31.1% [95% CI, 30.9%-31.2%), and men vs women (UHealth: 30.8% [95% CI, 30.4%-31.2%] vs 43.0% [95% CI, 42.6%-43.3%]; NYULH: 23.1% [95% CI, 22.9%-23.3%] vs 34.9% [95% CI, 34.7%-35.1%])—had significantly lower availability and comprehensiveness of cancer FHI (*P* < .001).

**Conclusions and Relevance:**

These findings suggest that systematic differences in the availability and comprehensiveness of FHI in the EHR may introduce informative presence bias as inputs to CDS algorithms. The observed differences may also exacerbate disparities for medically underserved groups. System-, clinician-, and patient-level efforts are needed to improve the collection of FHI.

## Key Points

**Question:**

What is the availability and comprehensiveness of family history information in electronic health records (EHRs) and how are these associated with clinical decision support algorithms?

**Findings:**

In this EHR quality improvement study that included 522 105 primary care patients, significant differences were found in family history availability and comprehensiveness based on sex, race and ethnicity, and language preference.

**Meaning:**

These findings suggest inadvertent exclusion of patients in historically medically underserved groups from identification by clinical decision support tools that depend on family history input, potentially further exacerbating or creating new health care disparities.

## Teaser

This quality improvement study examines disparities in the availability and comprehensiveness of cancer family history information by sex, race and ethnicity, and language preference in electronic health record data in 2 large US health care systems.

## Introduction

Clinical decision support (CDS) tools are increasingly used within health care systems to identify and treat patients in need of specialty services.^1,2,3,4,5^ Clinical decision support tools have been shown to expand the reach of such services to patients.^1,6,7,8,9^ However, recent ethical frameworks have highlighted that CDS tools can perpetuate unfair or biased practices in health care, inadvertently reinforcing or even creating health care disparities.^10,11^ These ethical frameworks assert that research should examine issues of fairness with regard to both individual-level and system-level factors in evaluating the impact of CDS tools and explicitly consider the impact on disparities in care.^10,12^ Normative analysis has shown that the integration of CDS algorithms needs to navigate multiple sources of potential bias, including bias in the underlying data, algorithm design, and delivery within health care settings.^12,13,14^ Algorithms that are based on electronic health record (EHR) data may be incomplete,^15^ reflecting patients’ interactions with the health care system,^13,16^ encoding health care inequalities into the data that serve as inputs for models.^17^ Incomplete EHR data can introduce a source of potential bias known as *informative presence bias* (ie, bias in the results of analyses based on EHR data due to systematic differences between data that are and are not observed owing to the structure of the observation process).^18,19^

One important source of input for CDS tools is family history information (FHI), because algorithms are increasingly being used to identify patients in need of services, such as cancer genetic services, based on their family history as an important marker for assessment of risk for multiple common conditions.^20,21,22^ However, FHI is often not adequately collected, recorded, or updated in a patient’s EHR.^23,24,25,26,27^ Less commonly assessed information such as diagnosis for second-degree relatives and age at disease diagnosis for all family members is important for risk stratification and is seldom collected.^27,28,29,30,31^ Resulting gaps in documentation of FHI in the EHR could be a critical source of informative presence bias that may differentially impact different patient subgroups. Although some prior research has investigated barriers to collection of FHI for medically underserved populations, this has often focused on what individual patients know about their family history.^32,33,34,35^ Differences in availability of FHI across patient subgroups within an EHR need further exploration.

Previous investigators^36,37,38^ have created a population health management approach that uses a standards-based CDS algorithm to identify unaffected patients eligible for genetic evaluation for hereditary breast, ovarian, prostate, pancreatic, and/or colorectal cancers based on cancer FHI available in the EHR and have demonstrated the feasibility of this approach. This CDS algorithm has the potential, in a given health care system, to identify thousands of patients in need of services. One critical issue in evaluating the development and implementation of system-level strategies is assessing whether an algorithm can exacerbate disparities already present in accessing cancer genetic services.^39,40,41,42,43^ Despite a strong interest in receiving services, individuals from racial and ethnic minority groups have disproportionately low access to and use of genetic services.^44,45,46,47,48^ Population health management approaches have the potential to address these disparities by broadly identifying eligible patients and offering genetic services. However, such strategies may also exacerbate existing disparities in cancer genetic services if the required FHI needed as inputs into the CDS algorithm are insufficient or completely missing to a disproportionate extent for some patient subgroups. To inform the potential impact of the implementation of our CDS algorithm on identification of patients, our research question was therefore to examine whether there are disparities in the availability and comprehensiveness of cancer FHI by sex, race, Hispanic or Latino ethnicity, and language preference in EHR data in 2 large US health care systems.

## Methods

### Settings

In this quality improvement study, we examined availability and comprehensiveness of cancer FHI available for cohorts of primary care patients within 2 health care systems: University of Utah Health (UHealth) and NYU Langone Health (NYULH). The UHealth system is one of the largest health care systems in the US Intermountain West region, providing care for 1.2 million residents of 6 states in a referral area that encompasses frontier (<7 persons per square mile), rural (<100 persons per square mile), and urban settings. The NYULH health system serves a diverse population of more than 8 million people receiving care at more than 300 ambulatory sites and affiliate hospitals anchored in the metropolitan New York City region. Sites were selected because they use the same EHR system (Epic Systems Corporation) supporting the CDS algorithm, but have different clinical structures, geographic locations in the US, and patient populations. The protocol was deemed a nonhuman participants quality improvement study by the institutional review boards of both sites; therefore, informed consent was not required. This study followed the Standards for Quality Improvement Reporting Excellence (SQUIRE) reporting guideline, when applicable.

### Eligible Patients

We examined the availability and comprehensiveness of FHI for all patients within the UHealth and NYULH systems who were between 25 and 60 years of age and had a primary care appointment during the previous 3 years. Data were collected or abstracted from the EHR from December 10, 2020, to October 31, 2021. These inclusion criteria were selected because this was the underlying patient population evaluated by the CDS algorithm.

### Data Abstracted

Using automated queries of the structured data within the family history section of the EHR at both health care systems, we examined the availability and comprehensiveness of family history generally and cancer family history specifically. Availability was defined as having any FHI and any cancer FHI in the EHR for a patient. For comprehensiveness, we focused on the presence or absence of 3 data elements needed for identification by the CDS algorithm in each observation of cancer family history in a patient’s EHR: specific type of cancer diagnosed in a family member, relationship of the family member to the patient (eg, mother, sister), and age at onset of cancer for the family member. Each observation captured cancer FHI about 1 condition from 1 specific family member. Data on sex, race and ethnicity, and language preference were also abstracted from structured EHR fields. In both health care systems, such demographic characteristics are generally either self-reported by patients on intake forms and entered into the EHR or collected through direct inquiry from front desk staff or medical assistants. In the EHR of both systems, predetermined categorizations have been created for sex (men and women) and race or ethnicity (Asian, Black, Hispanic or Latino, Native Hawaiian or other Pacific Islander, non-Hispanic or non-Latino, and White), each with options for “other.”

### Statistical Analysis

Data were analyzed from June 15 to October 31, 2021. Analyses of availability were conducted at the patient level, and analyses of comprehensiveness were conducted at the level of each observation of a family history of cancer. Descriptive statistics were completed using frequencies and percentages of the total numbers of patients or observations reviewed. Pearson χ^2^ tests were used to determine associations between FHI availability and demographics for patient-level analysis. Mixed-effects regression models were used to examine the bivariate associations between the 3 comprehensiveness indicators of family history and demographic characteristics at the observation level, accounting for the potential for multiple records for an individual patient. Analysis was conducted using Stata, version 17 (StataCorp LLC), and R, version 4.0.5 (R Project for Statistical Computing) with 2-sided *P* < .05 considered statistically significant.

## Results

### Availability of Any FHI in EHR

We first examined the availability of any FHI for the complete populations of 144 484 patients in the UHealth system (53.6% women and 36.5% men; 4.3% Asian, 2.4% Black, 1.4% Native Hawaiian or other Pacific Islander, and 67.6% White; 74.4% non-Hispanic or non-Latino; and 83.0% with an English language preference) and 377 621 patients in the NYULH system (55.3% women and 44.7% men; 7.0% Asian, 13.2% Black, 0.5% Native Hawaiian or other Pacific Islander, and 55.3% White; 63.2% non-Hispanic or non-Latino; and 89.9% with an English language preference) aged 25 to 60 years who had received primary care in the last 3 years. We found significant differences in the percentage of patients with any FHI in the EHR by sex, race, Hispanic or Latino ethnicity, and language preference in both health care systems (Table 1). Among primary care patients in the UHealth system, the proportion of patients with any FHI available differed significantly by race; this proportion was highest for White patients (70.8% [95% CI, 70.5%-71.1%]) and lowest for Black patients (54.3% [95% CI, 52.7%-56.0%]; *P* < .001). A higher proportion of non-Hispanic or non-Latino patients (69.5% [95% CI, 69.2%-69.8%]) had any FHI available compared with Hispanic or Latino patients (66.3% [95% CI, 65.6%-67.0%]; *P* < .001). A higher proportion of women had any FHI available (71.9% [95% CI, 71.6%-72.2%]) compared with men (64.3% [95% CI, 63.9%-72.2%]; *P* < .001). The proportion of English-speaking patients with any FHI available (70.0% [95% CI, 69.7%-70.2%]) was higher than that of Spanish-speaking patients (58.9% [95% CI, 57.7%-60.7%]; *P* < .001). Among primary care patients in the NYULH system, the proportion of patients with any FHI available also differed significantly by race; this proportion was highest for White patients (69.5% [95% CI 69.3%-69.7%]) and lowest for Native Hawaiian or other Pacific Islander patients (51.2% [95% CI, 46.5%-51.1%]; *P* < .001). A higher proportion of non-Hispanic or non-Latino patients (68.2% [95% CI, 68.0%-68.4%]) had any FHI available compared with Hispanic or Latino patients (63.9% [95% CI, 63.6%-64.2%]; *P* < .001). A higher proportion of women had any FHI available (71.7% [95% CI, 71.5%-71.9%]) compared with men (61.1% [95% CI, 60.9%-61.3%]; *P* < .001). The proportion of English-speaking patients with any FHI available (68.6% [95% CI, 68.4%-68.8%]) was higher than that of Spanish-speaking patients (51.9% [95% CI, 51.2%-52.6%]; *P* < .001).

**Table 1.** Availability of Any FHI in the EHR by Sex, Race and Ethnicity, and Language Preference

Characteristic	Any FHI available for patients, No. (%) [95% CI]					
	UHealth (n = 144 484)			NYULH (n = 377 621)		
	Yes (n = 103 694)	No (n = 40 790)	*P* value	Yes (n = 252 919)	No (n = 124 702)	*P* value
Sex						
Women	55 700 (71.9) [71.6-72.2]	21 801 (28.1) [27.8-28.4]	<.001	149 715 (71.7) [71.5-71.9]	58 988 (28.3) [28.1-28.5]	<.001
Men	33 933 (64.3) [63.9-64.7]	18 852 (35.7) [35.3-36.1]		103 162 (61.1) [60.9-61.3]	65 696 (38.9) [38.7-39.1]	
Not documented in EHR	14 061 (99.0) [98.9-99.2]	137 (1.0) [0.8-1.1]		42 (70.0) [57.0-80.4]	18 (30.0) [19.6-43.0]	
Race						
Asian	3900 (62.5) [61.3-63.7]	2339 (37.5) [36.3-38.7]	<.001	17 443 (65.7) [65.2-66.3]	9095 (34.3) [33.7-34.8]	<.001
Black	1922 (54.3) [52.7-56.0]	1615 (45.7) [44.0-47.3]		32 000 (64.1) [63.7-64.5]	17 933 (35.9) [35.5-36.3]	
Native Hawaiian or other Pacific Islander	1320 (64.0) [61.9-66.1]	742 (36.0) [33.9-38.1]		935 (51.2) [46.5-51.1]	892 (48.8) [48.9-53.5]	
White	69 107 (70.8) [70.5-71.1]	28 535 (29.2) [28.9-29.5]		145 139 (69.5) [69.3-69.7]	63 826 (30.5) [30.3-30.7]	
Other^a^	11 769 (64.9) [64.2-65.6]	6352 (35.1) [34.4-35.8]		27 117 (64.7) [64.3-65.2]	14 767 (35.3) [34.8-35.7]	
Not documented in EHR	15 694 (92.9) [92.5-93.2]	1207 (7.1) [6.8-7.5]		9175 (60.1) [59.3-60.8]	6103 (39.9) [39.2-40.7]	
Refused to answer	NA	NA		21 153 (63.7) [63.2-64.2]	12 043 (36.3) [35.8-36.8]	
Ethnicity						
Hispanic or Latino	12 733 (66.3) [65.6-67.0]	6475 (33.7) [33.0-34.4]	<.001	48 166 (63.9) [63.6-64.2]	27 211 (36.1) [35.8-36.4]	<.001
Non-Hispanic or non-Latino	74 698 (69.5) [69.2-69.8]	32 788 (30.5) [30.2-30.8]		162 842 (68.2) [68.0-68.4]	75 803 (31.8) [31.6-32.0]	
Not documented in EHR	16 263 (91.4) [91.0-91.8]	1527 (8.6) [8.2-9.0]		41 911 (65.9) [65.5-66.3]	21 688 (34.1) [33.7-34.5]	
Language preference						
English	83 890 (70.0) [69.7-70.2]	35 994 (30.0) [29.8-30.3]	<.001	232 869 (68.6) [68.4-68.8]	106 605 (31.4) [31.2-31.6]	<.001
Spanish	3606 (58.9) [57.7-60.2]	2513 (41.1) [39.8-42.3]		10 644 (51.9) [51.2-52.6]	9868 (48.1) [47.4-48.8]	
Other	16 198 (87.6) [87.2-88.1]	2283 (12.3) [11.9-12.8]		9406 (53.3) [52.6-54.1]	8229 (46.7) [45.9-47.4]	

Abbreviations: EHR, electronic health record; FHI, family history information; NA, not applicable; NYULH, NYU Langone Health; UHealth, University of Utah Health.

^a^ Selected if the patient did not meet any of the preestablished listed categorizations in the EHR systems.

### Availability of Cancer FHI

We found similar patterns of associations for availability of cancer FHI in the EHR (Table 2). Among primary care patients in the UHealth system, the proportion of patients with cancer FHI available differed significantly by race; this proportion was highest for White patients (42.8% [95% CI, 42.5%-43.1%]) and lowest for Black patients (17.3% [95% CI, 16.1%-18.6%]; *P* < .001). A higher proportion of non-Hispanic or non-Latino patients (40.2% [95% CI, 39.9%-40.5%]) had cancer FHI available compared with Hispanic or Latino patients (27.2% [95% CI, 26.5%-27.8%]; *P* < .001). A higher proportion of women had cancer FHI available (43.0% [95% CI, 42.6%-43.3%]) compared with men (30.8% [95% CI, 30.4%-31.2%]; *P* < .001). The proportion of English-speaking patients with cancer FHI available (40.0% [95% CI, 39.7%-40.3%]) was higher than that of Spanish-speaking patients (18.4% [95% CI, 17.2%-19.1%]; *P* < .001). Among primary care patients in the NYULH system, the proportion of patients with cancer FHI available differed significantly by race; this proportion was highest for White patients (33.8% [95% CI, 33.6%-34.0%]) and lowest for Native Hawaiian or other Pacific Islander patients (15.8% [95% CI, 13.2%-16.5%]; *P* < .001). A higher proportion of non-Hispanic or non-Latino patients (31.6% [95% CI, 31.4%-31.8%]) had cancer FHI available compared with Hispanic or Latino patients (24.4% [95% CI, 24.1%-24.7%]; *P* < .001). A higher proportion of women had cancer FHI available (34.9% [95% CI, 34.7%-35.1%]) compared with men (23.1% [95% CI, 22.9%-23.3%]; *P* < .001). The proportion of English-speaking patients with cancer FHI available (31.1% [95% CI, 30.9%-31.2%]) was higher than that of Spanish-speaking patients (15.1% [95% CI, 14.6%-15.6%]; *P* < .001). We observed the same patterns of associations among both patients with a personal cancer history and unaffected patients without a personal cancer history in stratified analyses, suggesting that the differences in availability of cancer FHI were not owing to underlying differences in cancer diagnoses between patient subgroups.

**Table 2.** Availability of Cancer FHI in the EHR by Sex, Race and Ethnicity, and Language Preference

Characteristic	Cancer FHI available for patients, No. (%) [95% CI]					
	UHealth patients (n = 144 484)			NYULH patients (n = 377 621)		
	Yes (n = 56 482)	No (n = 88 002)	*P* value	Yes (n = 111 774)	No (n = 265 847)	*P* value
Sex						
Women	33 291 (43.0) [42.6-43.3]	44 210 (57.0) [56.7-57.4]	<.001	72 822 (34.9) [34.7-35.1]	135 881 (65.1) [64.9-65.3]	<.001
Men	16 272 (30.8) [30.4-31.2]	36 513 (69.2) [68.8-69.6]		38 929 (23.1) [22.9-23.3]	129 929 (77.0) [76.7-77.1]	
Not documented in EHR	6919 (48.7) [47.9-49.6]	7279 (51.3) [50.4-52.1]		23 (38.3) [26.8-51.4]	37 (61.7) [48.6-73.2]	
Race						
Asian	1399 (22.4) [21.4-23.5]	4840 (77.6) [76.5-78.6]	<.001	6338 (23.9) [23.4-24.4]	20 200 (76.1) [75.6-76.6]	<.001
Black	611 (17.3) [16.1-18.6]	2926 (82.7) [81.4-83.9]		12 179 (24.4) [24.0-24.8]	37 754 (75.6) 75.2-76.0]	
Native Hawaiian or other Pacific Islander	483 (23.4) [21.6-25.3]	1579 (76.6) [74.7-78.4]		570 (15.8) [13.2-16.5]	3037 (84.2) [83.5-86.8]	
White	41 764 (42.8) [42.5-43.1]	55 878 (57.2) [56.9-57.5]		70 624 (33.8) [33.6-34.0]	138 341 (66.2) [66.0-66.4]	
Other^a^	4477 (24.7) [24.1-25.3]	13 644 (75.3) [74.7-75.9]		10 079 (25.1) [24.4-25.2]	30 025 (74.9) [74.8-75.6]	
Not documented in EHR	7748 (45.9) [45.1-46.6]	9135 (54.1) [53.4-54.9]		3485 (22.8) [22.1-23.5]	11 793 (77.2) [76.5-77.8]	
Refused to answer	NA	NA		8499 (25.6) [25.1-26.1]	4697 (74.4) [73.9-74.9]	
Ethnicity						
Hispanic or Latino	5219 (27.2) [26.5-27.8]	13 989 (72.8) [72.2-73.5]	<.001	18 366 (24.4) [24.1-24.7]	57 011 (75.6) [75.3-75.9]	<.001
Non-Hispanic or non-Latino	43 196 (40.2) [39.9-40.5]	64 290 (59.8) [59.5-60.1]		75 346 (31.6) [31.4-31.8]	163 299 (68.4) [68.2-68.6]	
Not documented in EHR	8067 (45.4) [44.6-46.1]	9723 (54.7) [53.9-55.4]		18 062 (28.4) [28.1-28.8]	45 537 (71.6) [71.2-71.9]	
Language preference						
English	47 970 (40.0) [39.7-40.3]	71 914 (60.0) [59.7-60.3]	<.001	105 471 (31.1) [30.9-31.2]	234 003 (68.9) [68.8-69.1]	<.001
Spanish	1108 (18.4) [17.2-19.1]	5011 (81.9) [80.9-82.8]		3098 (15.1) [14.6-15.6]	17 414 (84.9) [84.4-85.4]	
Other	7404 (40.1) [39.4-40.8]	11 077 (59.9) [59.2-60.6]		3205 (18.2) [17.6-18.8]	14 430 (81.8) [81.2-82.4]	

Abbreviations: EHR, electronic health record; FHI, family history information; NA, not applicable; NYULH, NYU Langone Health; UHealth, University of Utah Health.

^a^ Selected if the patient did not meet any of the preestablished listed categorizations in the EHR systems.

### Comprehensiveness of Cancer Family History Records

We next examined the comprehensiveness of cancer family history for 684 861 observations in the UHealth system and 1 154 161 observations in the NYULH system. We observed significant differences for each of the 3 data elements examined in both health care systems. Having the type of relative diagnosed with cancer specified in observations of cancer family history differed significantly by race for both UHealth and NYULH patients, with the proportion of observations with this information highest for White patients (17.4% [95% CI, 17.3%-17.5%] and 17.5% [95% CI, 17.4%-17.6%], respectively; *P* < .001) (Table 3). In addition, in both health care systems, the proportion of observations specifying the type of relative diagnosed with cancer was significantly higher for non-Hispanic or non-Latino (16.9% [95% CI, 16.8%-17.0%] at UHealth; 16.4% [95% CI, 16.3%-16.5%] at NYULH) compared with Hispanic or Latino patients (12.0% [95% CI, 11.8%-12.3%] at UHealth; 12.8% [95% CI, 12.7%-13.0%] at NYULH; *P* < .001 at both sites); for women (16.8% [95% CI, 16.7%-16.9%] at UHealth; 17.0% [95% CI, 17.0%-17.1%] at NYULH) compared with men (15.2% [95% CI, 15.1%-15.4%] at UHealth; 12.8% [95% CI, 12.7%-12.9%] at NYULH; *P* < .001 at both sites); and for English-speaking patients (16.7% [95% CI, 16.6-16.8] at UHealth; 15.8% [95% CI, 15.8%-15.9%] at NYULH) compared with Spanish-speaking patients (9.2% [95% CI, 8.7%-9.6%] at UHealth; 9.6% [95% CI, 9.3%-9.9%] at NYULH; *P* < .001 at both sites). We observed the same systematic differences for both health care systems by sex, race and ethnicity, and language preference as to whether observations specified the type of cancer with which the family member had been diagnosed (Table 4). We also generally observed the same systematic differences as to whether observations specified the age at onset of cancer for the family member, although the association between race and specifying age at onset was not significant in the UHealth system (Table 5). The number of observations specifying the age at onset was generally low for all groups in both systems.

**Table 3.** Presence of Information in Cancer Family History Observations in the EHR on Relative Diagnosed by Sex, Race and Ethnicity, and Language Preference

Characteristic	Type of relative specified in observations, No. (%) [95% CI]						
	UHealth (n = 684 861)				NYULH (n = 1 154 161)		
	Yes (n = 112 064)	No (n = 572 797)	*P* value	Yes (n = 178 395)		No (n = 975 766)	*P* value
Sex							
Women	70 734 (16.8) [16.7-16.9]	349 862 (83.2) [83.1-83.3]	<.001	122 503 (17.0) [17.0-17.1]		596 366 (82.9) [82.9-83.0]	<.001
Men	29 140 (15.2) [15.1-15.4]	162 332 (84.8) [84.6-84.9]		55 854 (12.8) [12.7-12.9]		379 224 (87.2) [87.1-87.3]	
Not documented in EHR	12 190 (16.7) [16.5-17.0]	60 603 (83.3) [83.0-83.5]		38 (17.8) [13.2-23.5]		176 (82.2) [76.5-86.8]	
Race							
Asian	2231 (11.3) [10.9-11.8]	17 457 (88.7) [88.2-89.1]	<.001	9399 (12.0) [11.7-12.2]		69 176 (88.0) [87.8-88.3]	<.001
Black	1050 (9.5) [9.0-10.0]	10 015 (90.5) [90.0-91.0]		18 564 (12.9) [12.7-13.0]		125 884 (87.1) [87.0-87.3]	
Native Hawaiian or other Pacific Islander	810 (10.8) [10.1-11.5]	6693 (89.2) [88.5-89.9]		362 (8.8) [8.0-9.7]		3754 (91.2) [90.3-92.0]	
White	86 736 (17.4) [17.3-17.5]	410 893 (82.6) [82.5-82.7]		113 606 (17.5) [17.4-17.6]		536 054 (82.5) [82.4-82.6]	
Other^a^	7495 (11.3) [11.1-11.6]	58 779 (88.7) [88.4-88.9]		17 334 (13.0) [12.8-13.2]		115 957 (87.0) [86.8-87.2]	
Not documented in EHR	13 742 (16.6) [16.4-16.9]	68 960 (83.4) [83.1-83.6]		5098 (12.9) [12.6-13.2]		34 426 (87.1) [86.8-87.4]	
Refused to answer	NA	NA		14 032 (13.4) [13.2-13.6]		90 515 (86.6) [86.4-86.8]	
Ethnicity							
Hispanic or Latino	8856 (12.0) [11.8-12.3]	64 756 (88.0) [87.8-88.2]	<.001	28 259 (12.8) [12.7-13.0]		191 844 (87.2) [87.0-87.3]	<.001
Non-Hispanic or non-Latino	88 830 (16.9) [16.8-17.0]	435 767 (83.1) [83.0-83.2]		121 729 (16.4) [16.3-16.5]		619 464 (83.6) [83.5-83.7]	
Not documented in EHR	14 378 (16.6) [16.3-16.8]	72 274 (83.4) [83.2-83.7]		28 407 (14.7) [14.6-14.9]		164 458 (85.3) [85.1-85.4]	
Language preference							
English	97 518 (16.7) [16.6-16.8]	486 723 (83.3) [83.2-83.4]	<.001	169 826 (15.8) [15.8-15.9]		903 522 (84.2) [84.1-84.2]	<.001
Spanish	1603 (9.2) [8.7-9.6]	15 890 (90.8) [90.4-91.3]		4319 (9.6) [9.3-9.9]		40 782 (90.4) [90.1-90.7]	
Other	12 943 (15.6) [15.3-15.8]	70 184 (84.4) [84.2-84.7]		4250 (11.9) [11.6-12.2]		31 461 (88.1) [87.8-88.4]	

Abbreviations: EHR, electronic health record; NA, not applicable; NYULH, NYU Langone Health; UHealth, University of Utah Health.

^a^ Selected if the patient did not meet any of the preestablished listed categorizations in the EHR systems.

**Table 4.** Presence of Information in Cancer Family History Observations in the EHR on Type of Cancer Diagnosed by Sex, Race and Ethnicity, and Language Preference

Characteristic	Type of cancer specified in observations, No. (%) [95% CI]					
	UHealth (n = 684 861)			NYULH (n = 1 154 161)		
	Yes (n = 71 394)	No (n = 613 467)	*P* value	Yes (n = 132 422)	No (n = 1 021 739)	*P* value
Sex						
Women	46 323 (11.0) [10.9-11.1]	374 273 (89.0) [88.9-89.1]	<.001	93 048 (12.9) [12.9-13.0]	625 821 (87.1) [87.0-87.1]	<.001
Men	17 002 (8.9) [8.8-9.0]	174 470 (91.1) [91.0-91.2]		39 345 (9.0) [9.0-9.1]	395 733 (91.0) [90.9-91.0]	
Not documented in EHR	8069 (11.1) [10.9-11.3]	64 724 (88.9) [88.7-89.1]		29 (13.6) [9.6-18.9]	185 (86.4) [81.1-90.4]	
Race						
Asian	1452 (7.4) [7.0-7.7]	18 236 (92.6) [92.3-93.0]	<.001	7150 (9.1) [8.9-9.3]	71 425 (90.9) [90.7-91.1]	<.001
Black	636 (5.7) [5.3-6.2]	10 429 (94.3) [93.8-94.7]		13 414 (9.3) [9.1-9.4]	131 034 (90.7) [90.6-90.9]	
Native Hawaiian or other Pacific Islander	466 (6.2) [5.7-6.8]	7037 (93.8) [93.2-94.3]		223 (5.4) [4.8-6.2]	3893 (94.6) [93.8-95.2]	
White	55 218 (11.1) [11.0-11.2]	442 411 (88.9) [88.8-89.0]		84 055 (12.9) [12.9-13.0]	565 605 (87.1) [87.0-87.1]	
Other^a^	4492 (6.8) [6.6-7.0]	61 782 (93.2) [93.0-93.4]		12 614 (9.5) [9.3-9.6]	120 677 (90.5) [90.4-90.7]	
Not documented in EHR	9130 (11.0) [10.8-11.3]	73 572 (89.0) [88.7-89.2]		3819 (9.7) [9.4-10.0]	35 705 (90.3) [90.0-90.6]	
Refused to answer	NA	NA		11 147 (10.7) [10.5-10.9]	93 400 (89.3) [89.1-89.5]	
Ethnicity						
Hispanic or Latino	5301 (7.2) [7.0-7.4]	68 311 (92.8) [92.6-93.0]	<.001	20 556 (9.3) [9.2-9.5]	199 547 (90.7) [90.5-90.8]	<.001
Non-Hispanic or non-Latino	56 573 (10.8) [10.7-10.9]	468 024 (89.2) [89.1-89.3]		89 585 (12.1) [12.0-12.2]	651 608 (87.9) [87.8-88.0]	
Not documented in EHR	9520 (11.0) [10.8-11.2]	77 132 (89.0) [88.8-89.2]		22 281 (11.6) [11.4-11.7]	170 584 (88.4) [88.3-88.6]	
Language preference						
English	61 953 (10.6) [10.5-10.7]	522 288 (89.4) [89.3-89.5]	<.001	127 036 (11.8) [11.8-11.9]	946 312 (88.2) [88.1-88.2]	<.001
Spanish	932 (5.3) [5.0-5.7]	16 561 (94.7) [94.3-95.0]		2457 (5.4) [5.2-5.7]	42 645 (94.6) [94.3-94.8]	
Other	8509 (10.2) [10.0-10.4]	74 618 (89.8) [89.6-90.0]		2929 (8.2) [7.9-8.5]	32 782 (91.8) [91.5-92.1]	

Abbreviations: EHR, electronic health record; NA, not applicable; NYULH, NYU Langone Health; UHealth, University of Utah Health.

^a^ Selected if the patient did not meet any of the preestablished listed categorizations in the EHR systems.

**Table 5.** Presence of Information in Cancer Family History Observations in the EHR on Age at Onset by Sex, Race and Ethnicity, and Language Preference

Characteristic	Age at onset specified in observations, No. (%) [95% CI]					
	UHealth (n = 684 861)			NYULH (n = 1 154 161)		
	Yes (n = 7698)	No (n = 677 163)	*P* value	Yes (n = 26 467)	No (n = 1 127 685)	*P* value
Sex						
Women	5456 (1.3) [1.3-1.3]	415 140 (98.7) [98.7-98.7]	<.001	21 288 (3.0) [2.9-3.0]	697 581 (97.0) [97.0-97.1]	<.001
Men	1517 (0.8) [0.8-0.8]	189 955 (99.2) [99.2-99.2]		5185 (1.2) [1.2-1.2]	429 893 (98.8) [98.8-98.8]	
Not documented in EHR	725 (1.0) [0.9-1.1]	72 068 (99.0) [98.9-99.1]		3 (1.4) [0.4-1.4]	211 (98.6) [95.7-99.6]	
Race						
Asian	223 (1.1) [1.0-1.3]	19 465 (98.9) [98.7-99.0]	.41	1678 (2.1) [2.0-2.2]	76 897 (97.9) [97.8-98.0]	<.001
Black	86 (0.8) [0.6-1.0]	10 979 (99.2) [99.0-99.4]		2892 (2.0) [1.9-2.0]	141 619 (98.0) [98.0-98.1]	
Native Hawaiian or other Pacific Islander	47 (0.6) [0.5-0.8]	7456 (99.4) [99.2-99.5]		69 (1.7) [1.3-2.1]	4047 (98.3) [97.9-98.7]	
White	5896 (1.2) [1.2-1.2]	491 733 (98.8) [98.8-98.8]		16 112 (2.5) [2.4-2.5]	633 548 (97.5) [97.5-97.6]	
Other^a^	626 (0.9) [0.9-1.0]	65 648 (99.1) [99.0-99.1]		3137 (2.3) [2.3-2.4]	130 154 (97.6) [97.6-97.7]	
Not documented in EHR	820 (1.0) [0.9-1.1]	81 882 (99.0) [98.9-99.1]		641 (1.6) [1.5-1.8]	38 883 (98.4) [98.2-98.5]	
Refused to answer	NA	NA		2010 (1.9) [1.8-2.0]	102 537 (98.1) [98.0-98.2]	
Ethnicity						
Hispanic to Latino	729 (1.0) [0.9-1.1]	72 883 (99.0) [98.9-99.1]	.02	4277 (1.9) [1.9-2.0]	215 826 (98.1) [98.0-98.1]	<.001
Non-Hispanic or non-Latino	6093 (1.2) [1.1-1.2]	518 504 (98.8) [98.8-98.9]		18 351 (2.5) [2.4-2.5]	722 842 (97.5) [97.5-97.6]	
Not documented in EHR	876 (1.0) [0.9-1.1]	85 776 (99.0) [98.9-99.1]		3848 (2.0) [1.9-2.1]	189 017 (98.0) [97.9-98.1]	
Language preference						
English	6754 (1.2) [1.1-1.2]	577 487 (98.8) [98.8-98.9]	<.001	25 247 (2.4) [2.3-2.4]	1 048 101 (97.6) [97.6-97.7]	<.001
Spanish	153 (0.9) [0.7-1.0]	17 340 (99.1) [99.0-99.3]		757 (1.7) [1.6-1.8]	44 345 (98.3) [98.2-98.4]	
Other	791 (0.9) [0.9-1.0]	82 336 (99.0) [99.0-99.1]		472 (1.3) [1.2-1.4]	35 239 (98.7) [98.6-98.8]	

Abbreviations: EHR, electronic health record; NA, not applicable; NYULH, NYU Langone Health; UHealth, University of Utah Health.

^a^ Selected if the patient did not meet any of the preestablished listed categorizations in the EHR systems.

### Demographic Characteristics of Patients Identified by the CDS Algorithm

The CDS algorithm identified 7340 patients (4.3%) from the underlying UHealth population and 21 913 (5.7%) from the underlying NYULH population. At both sites, the proportions of patients who were White, non-Hispanic or non-Latino, and women and who had an English language preference were higher among those identified by the CDS algorithm compared with those for the underlying patient populations (eTable in the Supplement).

## Discussion

Our analysis found systematic differences in the availability and comprehensiveness of FHI in the EHR for primary care patients in 2 large health care systems. Patients who were members of racial and ethnic minority groups had less available FHI—including cancer FHI—than White and non-Hispanic or non-Latino patients. Similarly, Spanish-speaking patients had less cancer FHI available and, when available, it was less comprehensive compared with that of English-speaking patients. These patterns strongly suggest that patients from demographic minority groups in medical care are less likely to be identified as needing specialty health care services or with tailored disease prevention recommendations if identification relies on FHI.

Our examination of the characteristics of patients identified by the CDS algorithm at both sites indicates that patterns of missing data contribute to differences in identification of patients eligible for cancer genetic evaluation. Family history documentation has been noted to be insufficient in two-thirds of EHRs in a primary care setting,^49,50^ and the present data show that this insufficiency has a disproportionate association with medically underserved groups. Findings such as these, which show the potential of an algorithm to exacerbate health disparities, are essential as part of continuous quality improvement efforts and allow solutions to be developed before integration into a health care system so that patients are not overlooked owing to missing or unavailable data.^51,52^ For example, automated procedures may be developed to circumvent potential biases owing to missing data.^53^ However, a recent review of generated prediction algorithms studies^54^ found only 54% of studies accounted for missing data in the EHR, and such solutions may fail to address issues of algorithmic fairness.^17^ Development of interventions to better collect FHI and potential development of targeted interventions to collect FHI from historically underserved groups and thereby address informative presence bias are also imperative. This work also highlights how the potential impact of new technologies on disparities should be embedded into the process of development and inform the decision to deploy based on the estimated impact on health inequities.^55^

Within the clinical context of inherited cancer, FHI drives other important decisions and recommendations in a health care system outside of the use of this information in a CDS algorithm. Family history can affect cancer screening recommendations.^56,57,58^ In addition, cancer FHI may help determine what type of genetic testing is ordered.^57^ Limited prior research has examined availability of FHI across patient subgroups.^59,60,61,62^ However, previous research has shown multiple barriers to the collection of FHI generally, including limited time, competing demands, reimbursement criteria, and clinician and staff training and knowledge.^63,64,65,66,67^ Prior studies have also shown underuse of FHI owing to incomplete or inaccurate information, lack of awareness about hereditary cancers, lack of awareness of evidence-based guidelines, and time constraints.^24,68,69^ The patterns observed herein strongly suggest at least 1 reason why underlying disparities in referral to and use of cancer genetic services may be systematic disparities in the collection of FHI in primary care clinics.^70^

Efforts have been launched to improve the collection of FHI through patient portals in EHR systems.^31,58,71^ The shift from patient intake forms to electronic formats has improved completeness, processing, updating, and storage of patient information,^31^ if patients have access to a patient portal system. As was seen in the transition to telehealth during the pandemic, the shift to digital FHI data collection may reinforce disparities driven by the digital divide.^72^ In addition, this puts the onus on patients to input comprehensive information about their own FHI, and patient-clinician discussions are often still needed to supplement patient input while addressing missing data.^31^ Thus, interventions for both patients and clinicians are needed to improve the collection of FHI across patient subgroups. Although FHI collection is taught in professional schools and emphasized in residency training for many clinicians, there are noted differences in clinical practice patterns across sites and specialties of care.^70,73,74,75,76^ Training and continuing education efforts with a cultural humility lens could address how to collect a complete family history that includes the elements needed to identify patients who may have inherited risk of disease,^77^ optimally combined with system-level prompts within the clinical workflow.

### Limitations

These findings should be considered in light of study limitations. The automated queries searched only structured EHR fields and did not include attachments or information documented in comments or narrative clinical notes. This could be especially relevant for patients that have “other” listed in their demographics fields but have notes that clarify the other categorizations.^78,79^ Therefore, patients who are members of sex or racial and ethnic minority groups could be missed by CDS algorithms. Clinicians in primary care may record FHI in different ways and may not use the structured fields. The large sample size enabled us to detect small differences with high power, and statistically significant differences should be considered in the context of the absolute differences between percentage estimates. Additionally, we are unable to determine the reasons for missing data in this analysis. For example, patients may have limited knowledge of FHI, potentially based on factors such as cultural norms around communication about disease conditions that vary across patient subgroups.^68,80^ Entry of FHI may have particular challenges for non–English-speaking populations; for example, FHI collection and discussion may be less extensive when interpreters are involved. Future research should assess the reasons for missingness of data and how missing data may affect identification by CDS algorithms.

## Conclusions

The findings of this quality improvement study suggest that there may be important systematic differences in the availability and comprehensiveness of cancer FHI by sex, race and ethnicity, and language preference in 2 large health care systems in different regions of the country with different structures. Such differences may exacerbate disparities in the identification of patients who require specialty services or tailored disease screening recommendations. System-level, clinician-level, and patient-level efforts are needed to improve the collection of FHI across subgroups, and efforts are particularly needed to improve availability and comprehensiveness of FHI for Black, Hispanic or Latino, Spanish-speaking, and male patients to mitigate the risk of health inequalities associated with these digital innovations.

## Notes

Supplement. **eTable.** Characteristics of Primary Care Patients Identified by the CDS Algorithm Compared With the Underlying Patient Populations
