# Supplementary material for: Association of Disparities in Family History and Family Cancer History in the Electronic Health Record With Sex, Race, Hispanic or Latino Ethnicity, and Language Preference in 2 Large US Health Care Systems
Source: JAMA Netw Open. 2022 Oct 4;5(10):e2234574. doi: 10.1001/jamanetworkopen.2022.34574 (PMC9533178; doi:10.1001/jamanetworkopen.2022.34574)
Supplement: Supplement. — eTable. Characteristics of Primary Care Patients Identified by the CDS Algorithm Compared With the Underlying Patient Populations [file jamanetwopen-e2234574-s001.pdf]

Supplementary Online Content

Chavez-Yenter D, Goodman MS, Chen Y, et al. Association of disparities in family history and family cancer history in the electronic health record with sex, race, Hispanic or Latino ethnicity, and language preference in 2 large US health care systems. *JAMA Netw Open*. 2022;5(10):e2234574. doi:10.1001/jamanetworkopen.2022.34574

eTable. Characteristics of Primary Care Patients Identified by the CDS Algorithm Compared With the Underlying Patient Populations

This supplementary material has been provided by the authors to give readers additional information about their work.

© 2022 Chavez-Yenter D et al. *JAMA Network Open*.

eTable. Characteristics of Primary Care Patients Identified by the CDS Algorithm Compared With the Underlying Patient Populations

	UHealth		NYU	
	**Primary care patients 25-60 years N=169,405**	**Identified by CDS algorithm N=7,340**	**Primary care patients 25-60 years N=385,854**	**Identified by CDS algorithm N= 21,913**
Met CDS algorithm criteria	7,340 (4.3%)	--	21,913 (5.6%)	--
**Race**				
Asian	7,906 (4.7%)	181 (2.5%)	23,590 (6.1%)	929 (4.2%)
Black	4,596 (2.7%)	116 (1.6%)	51,290 (13.3%)	2,082 (9.5%)
Native Hawaiian/ Pacific Islander	2,353 (1.4%)	67 (0.9%)	3,401 (0.9%)	78 (0.4%)
Other	24, 049 (14.2%)	754 (0.3%)	44,199 (11.5%)	2,024 (9.2%)
White	120,499 (71.1%)	6,098 (83.1%)	213,926 (55.4%)	14,364 (65.6%)
Not documented in EHR	3,131 (1.9%)	124 (1.7%)	12,283 (3.2%)	539 (2.5%)
Refused to answer	------	------	37,165 (9.6%)	1,897 (8.7%)
**Ethnicity**				
Hispanic	24,530 (14.5%)	798 (10.9%)	3,714 (1.0%)	323 (1.5%)
Non-Hispanic/Latino	140,650 (83.0%)	6,378 (86.9%)	27,802 (7.1%)	2,656 (12.1%)
Not documented in EHR	4,225 (2.5%)	164 (2.2%)	354,338 (91.8%)	18,934 (86.4%)
**Sex**				
Female	109,014 (64.4%)	6,179 (84.2%)	218,276 (56.6%)	16,230 (74.1%)
Male	60,391 (35.6%)	1,161 (15.8%)	167,578 (43.4%)	5,683 (25.9%)
**Language**				
English	155,263 (91.7%)	7,100 (96.7%)	344,781 (89.4%)	20,971 (95.7%)
Spanish	7,800 (4.6%)	162 (2.2%)	21,709 (5.6%)	363 (1.7%)
Other/Not documented in EHR	6,342 (3.7%)	78 (1.1%)	19,364 (5.0%)	579 (2.6%)

© 2022 Chavez-Yenter D et al. *JAMA Network Open*.
